# Gang truce for violence prevention, El Salvador

**DOI:** 10.2471/BLT.15.166314

**Published:** 2016-06-01

**Authors:** Charles M Katz, EC Hedberg, Luis Enrique Amaya

**Affiliations:** a Center for Violence Prevention & Community Safety, Criminology and Criminal Justice, Arizona State University Programs, 411 N. Central Street, Suite 680, Phoenix, Arizona 85004, United States of America (USA).; b NORC at the University of Chicago, Chicago, USA.; c Institute of Science, Technology and Innovation, Universidad Francisco Gavidia, San Salvador, El Salvador.

## Abstract

**Objective:**

To estimate the effects on homicide rates of the gang truce that was brokered in El Salvador in 2012.

**Methods:**

Mathematical models based on municipal-level census, crime and gang-intelligence data were used to estimate the effect of the truce on homicide rates. One model estimated the overall effect after accounting for the linear trend and seasonality in the homicide rate. In a moderated-effect model, we investigated the relationship between the truce effect and the numbers of MS13 (Mara Salvatrucha 13) and Eighteenth-Street gang members imprisoned per 100 000 population. We then ran each of these two models with additional control variables. We compared values before the truce – 1 January 2010 to 29 February 2012 – with those after the truce – 1 March 2012 to 31 December 2013.

**Findings:**

The overall-effect models with and without additional control variables indicated a homicide rate after the truce that was significantly lower than the value before the truce, giving rate ratios of 0.55 (95% confidence interval, CI: 0.49–0.63) and 0.61 (95% CI: 0.54–0.69), respectively. For any given municipality, the effectiveness of the truce appeared to increase as the number of MS13 gang members imprisoned per 100 000 population increased. We did not observe the same significant relationship for imprisoned Eighteenth-Street gang members.

**Conclusion:**

In the 22 months following the establishment of a national gang truce, the homicide rate was about 40% lower than in the preceding 26 months. The truce’s impact appeared particularly strong in municipalities with relatively high numbers of imprisoned MS13 gang members per 100 000 population.

## Résumé

**Objectif:**

Estimer les effets sur le taux d'homicide de la trêve des gangs qui a été signée au Salvador en 2012.

**Méthodes:**

Des modèles mathématiques établis d'après les recensements municipaux et les renseignements sur les gangs et la criminalité ont été utilisés pour estimer l'effet de la trêve sur le taux d'homicide. Un modèle a permis d'en estimer l'effet global après prise en compte de la tendance linéaire et de la saisonnalité du taux d'homicide. Un modèle à effets modérés nous a permis d'étudier la relation entre l'effet de la trêve et le nombre de membres emprisonnés des gangs MS13 (Mara Salvatrucha 13) et Barrio 18 pour 100 000 habitants. Nous avons ensuite soumis chacun de ces deux modèles à des variables de contrôle supplémentaire. Nous avons comparé les valeurs d'avant la trêve – du 1er janvier 2010 au 29 février 2012 – à celles d'après la trêve – du 1er mars 2012 au 31 décembre 2013.

**Résultats:**

Les modèles d'estimation de l'effet global, avec et sans variables de contrôle supplémentaire, ont indiqué un taux d'homicide après la trêve nettement inférieur aux valeurs d'avant la trêve, avec des ratios de taux respectifs de 0,55 (intervalle de confiance de 95%, IC: 0,49-0,63) et 0,61 (IC 95%: 0,54-0,69). Quelle que soit la municipalité, l'efficacité de la trêve grandissait au fur et à mesure que le nombre de membres emprisonnés du gang MS13 pour 100 000 habitants augmentait. Nous n'avons pas observé de rapport aussi significatif pour les membres emprisonnés du gang Barrio 18.

**Conclusion:**

Pendant les 22 mois qui ont suivi l'établissement d'une trêve nationale des gangs, le taux d'homicide était environ 40% plus faible que lors des 26 mois précédents. L'impact de la trêve était particulièrement marqué dans les municipalités où le nombre de membres emprisonnés du gang MS13 pour 100 000 habitants était relativement élevé.

## Resumen

**Objetivo:**

Estimar el impacto en las tasas de homicidio de la tregua entre bandas negociada en El Salvador en 2012.

**Métodos:**

Se utilizaron modelos matemáticos basados en un censo municipal, datos sobre crímenes e inteligencia de bandas para estimar el impacto de la tregua en las tasas de homicidio. Un modelo calculó el impacto general tras tener en cuenta la tendencia lineal y la estacionalidad de las tasas de homicidio. En un modelo de efecto moderado, se investigó la relación entre el efecto de la tregua y el número de miembros de las bandas MS13 (Mara Salvatrucha 13) y Calle 18 en prisión por cada 100 000 habitantes. Posteriormente, se implementaron ambos modelos con variables de control adicionales. Se compararon los valores antes de la tregua (del 1 de enero de 2010 al 29 de febrero de 2012) y los de después de la tregua (del 1 de marzo de 2012 al 31 de diciembre de 2013).

**Resultados:**

Los modelos de efecto general con y sin variables de control adicionales indicaron que la tasa de homicidio posterior a la tregua fue mucho menor que el valor antes de la misma, lo que ofrece coeficientes de la tasa del 0,55% (intervalo de confianza, IC, del 95%: 0,49-0,63) y del 0,61 (IC del 95%: 0,54-0,69), respectivamente. En todos los municipios, la eficacia de la tregua parecía aumentar a medida que aumentaba el número de miembros de la MS13 encarcelados por cada 100 000 habitantes. No se observó la misma relación significativa para los miembros de la Calle 18 encarcelados.

**Conclusión:**

Durante los 22 meses posteriores al establecimiento de una tregua nacional entre bandas, la tasa de homicidio fue un 40% inferior que en los 26 meses anteriores. El impacto de la tregua fue especialmente significativo en los municipios con un número relativamente alto de miembros de la MS13 encarcelados por cada 100 000 habitantes.

## ملخص

**الغرض:**

تقدير التأثيرات على معدل جرائم القتل نتيجة الهدنة بين العصابات التي تمت إقامتها في السلفادور عام 2012.

**الطريقة:**

تم استخدام نماذج رياضية تستند إلى البيانات المتعلقة بتعداد السكان على مستوى البلديات والجرائم والمعلومات الاستخبارية عن العصابات لتقدير تأثير الهدنة على معدلات جرائم القتل. قام نموذج واحد بتقدير التأثير الكلي بعد تفسير الاتجاه الخطي والتغيّرات الموسمية في معدل جرائم القتل. وفي أحد النماذج ذات التأثير المتوسط، قمنا بالتحقيق في العلاقة بين تأثير الهدنة وأعداد المسجونين من أفراد عصابة "إم إس 13" (مارتا ساڤاتروتشا 13) وعصابة "الشارع الثماني عشر" لكل 100,000 من السكان. ثم قمنا بعرض كلا هذين النموذجين مع المتغيرات الضابطة الإضافية. وقمنا بمقارنة القيم الصادرة قبل إقامة الهدنة – بدءًا من يوم 1 يناير/كانون الثاني 2010 حتى يوم 29 فبراير/شباط 2012 – مع تلك القيم الصادرة بعد إقامة الهدنة – بدءًا من يوم 1 مارس/آذار 2012 حتى يوم 31 ديسمبر/كانون الأول 2013.

**النتائج:**

إن النماذج ذات التأثير الكلي المزودة بالمتغيرات الضابطة الإضافية – أو غير المزودة بها – أشارت إلى أن معدل جرائم القتل التي حدثت بعد إقامة الهدنة كان أقل بكثير من قيمة معدل الجرائم قبل إقامة الهدنة، مما يدل على نسب معدلات تبلغ 0.55 (بنسبة أرجحية مقدارها ‏95%: 0.49–0.63) و0.61 (بنسبة أرجحية مقدارها 95‏%: 0.54–0.69)، على التوالي. وبالنسبة لأي من البلديات، فقد بدا أن تأثير الهدنة يزداد حيث ارتفع عدد المسجونين من عصابة "إم إس 13" لكل 100,000 من السكان. بينما لم نلحظ العلاقة ذات الدلالة الإحصائية نفسها للأفراد المسجونين من عصابة "الشارع الثماني عشر".

**الاستنتاج:**

كان معدل جرائم القتل أقل بحوالي ‏40% في الـ 22 شهرًا التالية لإقامة هدنة بين العصابات على المستوى الوطني، مما كان عليه في الـ 26 شهرًا السابقة. وبدا أن تأثير الهدنة قويًا بشكل خاص في البلديات التي تضم أعدادًا كبيرة نسبيًا من الأفراد المسجونين من عصابة "إم إس 13" لكل 100,000 من السكان.

## 摘要

**目的:**

旨在估计 2012 年在萨尔瓦多促成的帮派停战协议对凶杀率的影响。

**方法:**

我们使用依据市级普查、犯罪和帮派情报数据制定的数学模型估计停战协议对凶杀率的影响。 在考虑凶杀率的线性趋势和季节性之后，我们使用一种模型估计了总体影响。 在调整后的影响模型中，我们研究了停战协议的成效与每 100 000 人中入狱的救世鳟鱼帮 (MS13) 和十八街黑帮成员人数之间的关系。 然后，我们采用其他控制变量分别运行了这两个模型。 我们比较了停战协议之前的数值——2010 年 1 月 1 日至 2012 年 2 月 29 日——与停战协议之后的数值——2012 年 3 月 1 日至 2013 年 12 月 31 日。

**结果:**

采用和不采用其他控制变量的总体效应模型表明停战协议之后的凶杀率显著低于停战协议之前，比率分别达到 0.55（95％ 置信区间，CI: 0.49–0.63） 和 0.61 (95% CI: 0.54–0.69)。 对于任何指定的直辖市来说，由于每 100 000 人中入狱的 MS13 帮成员人数的增加，停战协议的成效也随之呈现出提高。 我们没有观察到入狱的十八街黑帮成员呈现出同等显著程度的关系。

**结论:**

在全国帮派停战协议签订之后的 22 个月内，凶杀率相较于之前的 26 个月降低了 40% 左右。 在每 100 000 人中入狱的 MS13 帮成员人数相对较高的直辖市中，停战协议的成效尤为显著。

## Резюме

**Цель:**

Дать оценку влияния перемирия между бандами, заключенного в Сальвадоре в 2012 году, на показатели числа убийств.

**Методы:**

Оценка влияния перемирия на показатели числа убийств была дана с помощью математических моделей на основе переписных данных муниципального уровня, данных о преступности и разведывательных данных о бандах. С помощью одной модели общее влияние рассчитывалось после учета линейного тренда и сезонных колебаний показателей числа убийств. В модели модифицированного влияния были изучены связи между влиянием перемирия и количеством членов банды MS13 (Мара Сальватруча 13 (Mara Salvatrucha 13)) и банды 18-й улицы (Eighteenth-Street), заключенных в тюрьму, из расчета на 100 000 жителей. Затем обе эти модели были реализованы с дополнительными контрольными переменными. Значения, относящиеся к периоду до перемирия (от 1 января 2010 года до 29 февраля 2012 года), были сравнены со значениями, относящимися к периоду после перемирия (от 1 марта 2012 года до 31 декабря 2013 года).

**Результаты:**

Реализация моделей общего влияния с дополнительными контрольными переменными и без них показала, что уровни числа убийств после перемирия были значительно меньше значений до него. В результате были получены отношения рисков, равные 0,55 (95%-й доверительный интервал, ДИ: 0,49–0,63) и 0,61 (95%-й ДИ: 0,54–0,69) соответственно. Для каждого муниципального образования было выявлено, что эффективность перемирия напрямую зависела от того, сколько членов банды MS13, заключенных под стражу, приходилось на 100 000 жителей. Такая же заметная связь с количеством заключенных под стражу членов банды 18-й улицы не наблюдалась.

**Вывод:**

Через 22 месяца после заключения национального перемирия между бандами показатели убийств снизились приблизительно на 40% по сравнению с предыдущими 26 месяцами. Влияние перемирия оказалось особенно сильным в муниципальных образованиях с относительно большим количеством заключенных в тюрьму членов банды MS13 (из расчета на 100 000 жителей).

## Introduction

In 2012, there were 16.3 homicides per 100 000 population in the Americas as a whole and 26.5 per 100 000 in Central America.^1^ Gang-related violence accounts for an estimated 70% of the homicides in El Salvador and for similarly large proportions of the homicides in Honduras and Guatemala.^2^ Some academics have attributed the relationship between gangs and violence to the collective and normative structure of gangs, which encourages and supports the use of both preemptive and retaliatory violence.^3^ When gang culture assumes violence to be the only effective response to actual or perceived threats and attacks, the result is spiralling inter-gang conflict and escalating violence.^3^ For example, a perceived slight violation of a gang’s turf or another sign of apparent disrespect may trigger a shooting that evokes a retaliatory shooting that, in turn, elicits another retaliatory shooting.^4^

In general, policy-makers have relied on the police to respond to violent gang conflicts that result in homicides. However, this practice has several shortcomings. First, gang members are unlikely to contact the police to resolve a conflict when doing so would result in loss of status and possible exposure of illegal activities to the police.^5^ Second, the residents of neighbourhoods with gang problems are often reluctant to call the police for fear of gang reprisal^6^ and often have a poor perception of the police.^7^ Third, the police typically operate by responding reactively to specific incidents rather than proactively to the ongoing problem of disputes between gangs.^8^

Some policy-makers and community activists have suggested the gang truce as an alternative to reliance on the police.^9^ As a nonviolent resolution to larger conflicts between gangs, the gang truce can have an impact on general levels of violence and other forms of criminality within communities.^10^^–^^12^ The goal of such a truce is to reduce or even eliminate violent conflict between gangs, by renegotiating existing norms within and between the gangs.^13^ As a violent dispute escalates between gangs, any leaders and members who do not respond with an appropriate amount of force risk appearing weak both to members of their own gang and to members of rival gangs.^13^ As the cycle of violence escalates between gangs, behavioural norms shift towards the increased valuation of violence for resolving conflicts – because violence becomes the most readily available and widely understood option.^13^ A gang truce, which is often mediated by a third party, is believed to be capable of breaking such a cycle of violence – in part, by providing the disputing gangs with a cooling-off period during which different, less harmful norms of expected behaviour may be established within and between the gangs.^9^^,^^13^

There has been little research on the effectiveness of gang truces. Studies of a truce, in south-central Los Angeles, United States of America, made between the Crips and the Bloods gangs in 1992, revealed temporary reductions in the number of homicides and gunshot wounds during the truce.^10^^,^^14^ However, the initial reports of these studies failed to point out that, although there was a 35% decrease in the homicide rate for the first three months of the truce, that rate doubled in the following eight months.^11^ A similar boomerang effect was reported in a study of a gang truce in Trinidad and Tobago.^15^ It appears that gang truces may produce short-term benefits but have adverse consequences in the long term. In this article, we report the results of the first large-scale quasi-experimental study of a government-facilitated gang truce that was designed to reduce the number of homicides.

## Methods

### Setting

We analysed data from El Salvador, where homicide is defined as a death deliberately inflicted on a person by another person.^1^ Between 2000 and 2011, the homicide rate in El Salvador increased from 39.3 to 69.2 per 100 000 population^1^ and El Salvador became one of the most violent nations in the world.^16^ About 44% of the country’s homicides occur in just 10 (3.8%) of the country’s 262 municipalities.^2^

In early 2012, members of the national government, then headed by President Mauricio Funes, led a group of negotiators, including a former United States congressman and members of the Catholic Church and the Organization of the American States, in framing the conditions for a truce between the MS13 (Mara Salvatrucha 13) and Eighteenth-Street gangs – that is, the two gangs that, together, represent 99% of gang members in El Salvador.^17^ By March 2012, an agreement between this group and representatives of the gangs had been reached. The primary goal of the gang truce was to reduce violence in general and homicides in particular. The terms, which were all implemented for both gangs, included that, in exchange for the gangs acting to reduce homicides, certain imprisoned gang members would be transferred to lower-security prisons, receive special visitation privileges and be permitted communication with those outside the prison – so that they could conduct crisis interventions to mitigate gang violence.^18^ Gang leaders also agreed to stop recruiting children, reduce violence against women and continue to participate in further negotiations.^19^

### Data

We examined the impact of the gang truce by linking four data sets. First, data from the Salvadorian Ministry of the Economy’s 2007 population and housing census provided municipal-level measures of community social structure. Second, municipal-level crime data for extortion, homicide, rape, robbery, theft, vehicle theft and vehicle robbery were provided – by month for the period between 1 January 2010 and 30 June 2014 – by the national civil police force. Data on the disappearance of individuals, aggregated by year and municipality, were also provided by the police. We examined the reliability of the police homicide data by comparing it with the homicide data collected, independently, by the Salvadorian Institute of Legal Medicine, which is a forensic science centre; for the other crime types of interest, we had no means to assess the extent of unreporting. Third, estimates of the numbers of MS13 and Eighteenth-Street gang members present in each municipality in 2011 were provided by the police. Last, we acquired, from the Directorate General of Prisons, the numbers of MS13 and Eighteenth-Street members imprisoned in each municipality in 2011. In 2011, about 55.6% of prisoners in El Salvador were members of one of these two gangs.^2^

The municipal–month was our primary unit of analysis. As we had 54 months of data for each of the 262 municipalities in the nation, we had 12 576 data points. The official crime data were collected for the 26 months (1 January 2010 to 29 February 2012) before the implementation of the gang truce and the 22 months (1 March 2012 to 31 December 2013) that immediately followed the implementation. The variables listed in Table 1 were used as controls in two of our mathematical models because they have consistently been associated with both violence and gang violence.^20^ Our measure of ethnic heterogeneity, which could vary from 0 to 1, was calculated by subtracting the sum of the squared proportions of the population in each ethnic group from a value of 1.^21^

**Table 1:** Descriptive statistics of data included in the gang truce study, El Salvador, 2010–2013

Variable	National value	Mean (SD) municipal value (*n* = 262)
**% of households**		
Rented	13.0	8.0 (4.8)
Urban	62.7	39.7 (24.6)
**% of population**		
Born in another municipality	23.7	18.9 (12.2)
Educated to at least secondary level	12.6	7.4 (6.6)
In female-headed household	34.8	34.3 (5.0)
Male aged 10–29 years	19.1	19.6 (1.7)
Unemployed	11.6	11.8 (6.9)
**Gang representation**		
Eighteenth-Street		
No. of prisoners	3323.0	12.7 (41.0)
No. of prisoners per 100 000 residents	57.9	28.7 (57.9)
No. of members on the street	6585.0	25.1 (86.2)
No. of members on the street per 100 000 residents	114.6	57.9 (160.7)
MS13		
No. of prisoners	4139.0	15.8 (39.6)
No. of prisoners per 100 000 residents	72.1	44.7 (51.4)
No. of members on the street	11 000	41.5 (106.5)
No. of members on the street per 100 000 residents	189.1	114.6 (204.6)
**Municipality participation in VFMP**	0.1	0.0 (0.2)
**Per-capita income, US$**	789.2	546.4 (265.4)
**Population**	5 744 113	22 000 (38 000)
**Racial/ethnic heterogeneity**	0.2	0.7 (0.1)

SD: standard deviation; US$: United States dollars; VFMP: Violence-free Municipality Programme.

We included, as count variables, the numbers of MS13 and Eighteenth-Street gang members who were on the streets and in prison within each municipality. Our aim was to see if the impact of the truce was affected by the level of gang presence – i.e. to see if municipalities with high numbers of MS13 and Eighteenth-Street gang members, whether in prison or on the street, experienced a relatively large reduction in the homicide rates because the two gangs had a greater span of control in such municipalities. We controlled for the observed spatial clustering in the number of imprisoned MS13 and Eighteenth-Street members by including the spatial lag of such gang members in our final models.

To estimate the effect of the truce, we used a predictor variable coded as 0 or 1 – to represent, respectively, the pre-truce and post-truce months for each municipality. Eleven municipalities had participated in a separate initiative, the Violence-Free Municipality Programme, in which gang members agreed to stop violence in exchange for a reduction in police operations.^22^ We included a dummy variable for each of these communities, for all months.

### Analysis

We investigated trends in the homicide rates and conducted a simple *t*-test comparison of the mean numbers – per 100 000 population per month – of homicides and of each of the other types of crime we investigated in the study periods before and after the truce. We created a cross-sectional time-series model to estimate the overall effect of the truce after accounting for linear trends and seasonality. We then conducted a detailed analysis of the truce’s effects, at the municipal level, using four mixed-effect negative binomial regression models. All four models used a population offset effect and included temporal and spatial lags to control for autocorrelation. The first model estimated an overall effect after accounting for linear trends and seasonality. The second model investigated how the truce effect was moderated by inclusion of natural logs of the number of MS13 or Eighteenth-Street gang members imprisoned per 100 000 population. Each of these two models was then rerun with the inclusion of additional control variables (Table 1). Due to space limitations and the fact that many of the control variables were not found to be statistically significant, we do not report the full regression models. However, the effects of the truce and the effects moderated by municipal-level gang presence in the prisons were found to be significant and are therefore reported as the ratios of the rates of homicide before the truce to the corresponding rates after the truce was agreed.

In our final analysis we considered two types of displacement: displacement by crime type and displacement by crime method. Crime-type displacement occurs when offenders who focus on one form of crime switch to another form of crime to avoid detection or to benefit in some other way.^23^ Crime-method displacement occurs when, because of an intervention, offenders change their tactics or methods of conducting crimes.^23^ Some critics of the Salvadorian gang truce have suggested that as homicides decreased, other forms of criminality – e.g. extortion – might have increased because of gang members’ increased freedom to conduct activities inside and outside prison.^16^ Others have argued that gang members hid the bodies of homicide victims to avoid detection and to protect the integrity of the country’s gang truce.^24^

## Results

Analysis showed no significant difference between the homicide data provided by the police and that collected by the Institute of Legal Medicine. The level of correlation between the two data sets was found to be 0.995.

**Fig. 1:**
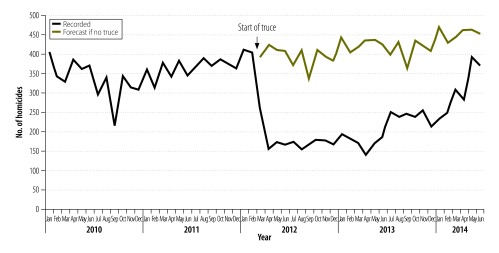
Monthly numbers of homicides, El Salvador, 2010–2014

Note: The plot shows the number of homicides recorded by the Salvadorian national civil police force and, for the period after the truce was agreed, the numbers of homicides that might have been expected if the trends recorded before the truce had remained unchanged.

The mean number of monthly homicides declined from about 354 before the truce to about 218 following the truce, giving a net decrease of about 136 homicides per month. Fig. 1 presents the number of police-recorded homicides in El Salvador over the study period. The period right after the start of the truce shows a sharp decline in the recorded homicide rate. When we compared a prediction of the number of homicides in the truce period, based on the data before the truce, with the number of homicides recorded in the same period, we found that the truce had prevented about 5500 homicides – assuming that all other relevant factors remained constant.

Table 2 presents the mean values and standard deviations for the numbers and rates – per 100 000 population – of disappearances, extortions, homicides, robberies, rapes, thefts and vehicular crimes for the municipal-months before and after the truce began. Based on these mean values, it appears that, after the truce was agreed, the rates of homicide and extortion were significantly lower than before the truce (*P* < 0.001) whereas the rate of rape was significantly higher (*P* = 0.012). However, as the mean values fail to capture any trends before the truce, they do not allow the significant differences to be attributed to the truce.

**Table 2:** Levels of crime before and after the gang truce, El Salvador, 2010–2013

Crime	Mean (SD) monthly value per municipality (*n* = 262)		Difference in means**^a^**	*P***^b^**	Autoregressive models^c^	
	Pre-truce**^d^**	Post-truce**^e^**			Difference	*P*
No. of disappearances	3.01 (10.39)	3.31 (8.20)	0.30	0.32	−2.87	0.00
No. of disappearances per 100 000 residents	8.07 (15.32)	9.35 (13.00)	1.27	0.10	−9.03	0.00
No. of extortions	1.16 (3.97)	0.90 (3.20)	−0.26	0.00	0.00	0.92
No. of extortions per 100 000 residents	3.47 (7.69)	2.65 (6.63)	−0.81	0.00	−0.01	0.71
No. of homicides	1.35 (3.16)	0.74 (1.76)	−0.61	0.00	−0.07	0.00
No. of homicides per 100 000 residents	4.21 (8.03)	2.86 (6.55)	−1.4	0.00	−0.22	0.00
No. of rapes	0.11 (0.42)	0.12 (0.43)	0.01	0.11	0.00	0.99
No. of rapes per 100 000 residents	0.50 (2.74)	0.66 (3.78)	0.16	0.01	0.00	0.99
No. of robberies	1.77 (8.11)	1.73 (7.10)	−0.05	0.46	0.00	0.86
No. of robberies per 100 000 residents	5.36 (9.67)	5.47 (9.46)	0.12	0.55	0.00	1.00
No. of thefts	3.25 (12.42)	3.13 (11.71)	−0.12	0.06	0.00	0.87
No. of thefts per 100 000 residents	12.32 (17.15)	11.98 (16.77)	−0.34	0.29	−0.01	0.95
No. of vehicle robberies or thefts	1.16 (8.06)	1.13 (7.36)	−0.03	0.58	0.00	0.97
No. of vehicle robberies or thefts per 100 000 residents	1.71 (12.59)	1.59 (5.35)	−0.12	0.79	0.00	0.95

SD: standard deviation.

^a^ Post-truce mean minus pre-truce mean.

^b^ From a *t*-test corrected for correlated observations within municipalities

^c^ Corrected for correlated observations within municipalities, autocorrelation across time and trend and heteroskedastic effects across municipalities.

^d^ 1 January 2010 – 29 February 2012.

^e^ 1 March 2012 – 31 December 2013.

In our basic regression models, which we used to adjust for any trends before the truce, we still found that the rates of homicide per 100 000 population – but not those of any other crime we investigated – were significantly lower after the truce than before (*P* = 0.002; Table 2). As a result, we used a more detailed model to analyse the possible effect of the truce on homicide rates. Inclusion in the overall-effect model of adjustments for multiple potential confounders reduced our calculated rate ratios – for the homicide rate per 100 000 population – from 0.61 (95% confidence interval, CI: 0.54–0.69) to 0.55 (95% CI: 0.49–0.63; Table 3; available at: http://www.who.int/bulletin/volumes/94/9/15-166314). 

**Table 3:** Homicide rate ratios derived from statistical models, El Salvador, 2010–2013

Model**^a^**	Homicide rate ratio (95% CI)**^b^**	
	Without controls	With controls**^c^**
**Overall effect**	0.61 (0.54–0.69)	0.55 (0.49–0.63)
**Moderated effects**^d^		
No. of imprisoned Eighteenth-Street gang members per 100 000 residents		
10	1.14 (0.91–1.43)	1.13 (0.90–1.43)
50	1.23 (0.95–1.60)	1.22 (0.94–1.59)
100	1.27 (0.96–1.68)	1.26 (0.96–1.67)
200	1.31 (0.97–1.78)	1.31 (0.97–1.76)
400	1.36 (0.98–1.88)	1.35 (0.98–1.87)
No. of imprisoned MS13 gang members per 100 000 residents		
10	0.62 (0.53–0.72)	0.61 (0.52–0.72)
50	0.45 (0.37–0.54)	0.44 (0.37–0.53)
100	0.39 (0.32–0.48)	0.38 (0.31–0.47)
200	0.34 (0.27–0.42)	0.33 (0.27–0.42)
400	0.29 (0.23–0.38)	0.29 (0.22–0.37)

CI: confidence interval.

^a^ All models were mixed-effect negative binomial regressions with a population offset, a random intercept and random slope for the effect of the truce on homicide rates, a two-month lag and a spatial lag on the homicide rate, a linear time trend and indicators for the calendar month.

^b^ The ratio of the number of homicides per 100 000 residents in the period before the truce – i.e. 1 January 2010 to 29 February 2012 – to the corresponding number in the period after the truce – i.e. 1 March 2012 to 31 December 2013.

^c^ Controls included the percentages of residents educated to at least secondary school level, unemployed, male and aged 10–29 years, living in a female-headed household and/or born in another municipality, per-capita income, the percentage of households that were in urban areas and/or rented, measures of racial/ethnic heterogeneity and municipality participation in the Violence-free Municipality Programme and, for each gang, log_e_(number of imprisoned gang members per 100 000 residents), log_e_(number of gang members on the street per 100 000 residents) and spatial lags.

^d^ All moderated models included, for each gang, log_e_(number of imprisoned gang members per 100 000 residents), log_e_(number of gang members on the street per 100 000 residents) and spatial lags.

The moderation coefficient for the interaction between the truce and the municipal-level rate of imprisonment of gang members per 100 000 population was only significant for the MS13 gang – not for the Eighteenth-Street. We found that the effectiveness of the truce, in reducing the homicide rate in a municipality, increased as the number of imprisoned MS13 gang members per 100 000 residents of that municipality increased. For example, in a municipality with 10 imprisoned MS13 gang members per 100 000 residents, the effect of the intervention was similar to the overall effect. In municipalities with 400 imprisoned MS13 gang members per 100 000 residents, however, there appeared to be a 70% reduction (Table 3 available at: http://www.who.int/bulletin/volumes/94/9/15-166314) in the corresponding homicide rate. This pattern remained substantially similar whether we made adjustments for multiple potential confounders or not.

We found no evidence of displacement by crime type or crime method. When temporal trends were included in the autoregressive models, we found no significant difference between the pre-truce rates of extortion, rape, robbery, theft or vehicular crime and the corresponding rates after the truce. In the period after the truce was agreed, annualized rates of disappearance appeared slightly higher than in the pre-truce period. However, the difference in rates did not appear to be statistically significant when investigated in a *t*-test (Table 2). Furthermore, analysis of the same data in a more complex autoregressive model with adjustment for temporal trends indicated that the gang truce had not caused an increase but a significant decrease in the rate of disappearance (Table 2).

## Discussion

Our findings indicate that – even after controlling for potentially confounding municipal-level socioeconomic variables, the presence of other gang interventions and spatial lags in gang presence – El Salvador’s gang truce significantly reduced the homicide rate and did not result in significant displacement by crime type or method. Our findings also show that the truce had a particularly beneficial effect on homicides in the municipalities that had relatively high numbers of imprisoned MS13 gang members. Prior research has indicated that imprisoned members of the MS13 gang have substantial influence over violence in Salvadorian communities^25^ – perhaps even more than the police and courts.^26^ MS13 is perhaps one of the most organizationally sophisticated street gangs in the Western Hemisphere.^26^ It has a vertical organizational structure and strong control over criminal enterprises in gang-controlled neighbourhoods and, although much of its leadership is imprisoned, it remains capable of enforcing its own rules.^27^ Our findings indicate that imprisoned MS13 gang leaders retain a strong influence over more junior gang members on the street. Although MS13 and Eighteenth-Street may be generally similar,^26^ there was a conflict between two factions within Eighteenth-Street during our study period (G Perseu, personal communication, 2014) and this may have weakened the gang’s leadership structure – to the point that the imprisoned leaders may have had less influence over violence by members on the street than their counterparts in MS13.

Unfortunately, the homicide rate began to increase slowly about 12 months after the truce was agreed and had approached levels recorded before the truce after a further 16 months. In 2014 a new government announced that imprisoned gang leaders would no longer receive any benefits of the truce. Following this declaration – and after our study period – homicide rates in El Salvador increased to record highs.^28^ It seems possible that the truce may have increased gang cohesion. It has been suggested that the government officials who negotiate gang truce might inadvertently be acknowledging gangs as legitimate social entities.^29^ By increasing cohesion within gangs, this may, in the long term at least, lead to increased levels of criminality.^29^^,^^30^ In some developing nations, gang leaders and elected officials may have similar levels of power and authority.^31^^–^^33^

In conclusion, we found that the Salvadorian gang truce agreed in March 2012 had a beneficial but short-lived impact on the homicide rate. However, the truce lasted longer than any other successfully negotiated gang truce.^11^^,^^15^ The Salvadorian truce appeared so successful that several other countries – e.g. Belize, Guatemala and Honduras – have sought to replicate it.^34^^,^^35^ In the future, before evaluating whether a truce strategy might be appropriate in a country, policy-makers should evaluate whether the conditions that allowed short-term success of the gang truce in El Salvador exist in other violent areas. They should also be warned that the potential for long-term negative consequences might outweigh any potential short-term benefits.
